# Localization and Distribution of '*Candidatus* Liberibacter asiaticus’ in Citrus and Periwinkle by Direct Tissue Blot Immuno Assay with an Anti-OmpA Polyclonal Antibody

**DOI:** 10.1371/journal.pone.0123939

**Published:** 2015-05-06

**Authors:** Fang Ding, Yongping Duan, Cristina Paul, Ronald H. Brlansky, John S. Hartung

**Affiliations:** 1 USDA ARS Molecular Plant Pathology Laboratory, Beltsville, Maryland, United States of America; 2 College of Plant Science and Technology, Huazhong Agricultural University, Wuhan, P. R. China; 3 USDA ARS Horticultural Research Laboratory, Fort Pierce, Florida, United States of America; 4 University of Florida, citrus Research and Education Center, Lake Alfred, Florida, United States of America; Shanghai Jiao Tong University, CHINA

## Abstract

‘*Candidatus* Liberibacter asiaticus’ (CaLas), a non-cultured member of the α-proteobacteria, is the causal agent of citrus Huanglongbing (HLB). Due to the difficulties of in vitro culture, antibodies against CaLas have not been widely used in studies of this pathogen. We have used an anti-OmpA polyclonal antibody based direct tissue blot immunoassay to localize CaLas in different citrus tissues and in periwinkle leaves. In citrus petioles, CaLas was unevenly distributed in the phloem sieve tubes, and tended to colonize in phloem sieve tubes on the underside of petioles in preference to the upper side of petioles. Both the leaf abscission zone and the junction of the petiole and leaf midrib had fewer CaLas bacteria compared to the main portions of the petiole and the midribs. Colonies of CaLas in phloem sieve tubes were more frequently found in stems with symptomatic leaves than in stems with asymptomatic leaves with an uneven distribution pattern. In serial sections taken from the receptacle to the peduncle, more CaLas were observed in the peduncle sections adjacent to the stem. In seed, CaLas was located in the seed coat. Many fewer CaLas were found in the roots, as compared to the seeds and petioles when samples were collected from trees with obvious foliar symptoms. The direct tissue blot immuno assay was adapted to whole periwinkle leaves infected by CaLas. The pathogen was distributed throughout the lateral veins and the results were correlated with results of qPCR. Our data provide direct spatial and anatomical information for CaLas in planta. This simple and scalable method may facilitate the future research on the interaction of CaLas and host plant.

## Introduction

Huanglongbing (HLB), also known as citrus greening, is considered the most devastating disease of citrus, and is widely distributed in more than 40 countries in Asia, Africa and America [1, 2]. HLB threatens the citrus industry in Asia where it has long been endemic and in citrus growing areas, such as Brazil and Florida, and where the disease was confirmed in 2004 and 2005. The disease was first unambiguously described in India but the symptoms were attributed to damage from psyllids [3] and the disease was referred to as ‘dieback’ in the central provinces of India in the late 19th century [4–6]. Effective therapeutic treatments, or resistant cultivars of citrus are not available for HLB, although thermal therapy [7] and tolerant rootstocks are being tested [8], and nutritional supplementation and rigorous control of psyllids can prolong the productive life of groves in Florida [9, 10]. Once CaLas has infected a plant, yellow shoots are produced which develop leaves with a blotchy mottle. Fruits may be malformed with color inversion. Leaf and fruit drop and shoot dieback are part of a subsequent decline and greatly shortens the lifespan of citrus trees [2, 11, 12]. Three species of bacteria are associated with HLB: ‘*Ca*. Liberibacter asiaticus’ (CaLas), ‘*Ca*. L. africanus’ (CaLaf), and ‘*Ca*. L. americanus’ (CaLam) [2, 13] though CaLas is the only species with a global distribution [14]. CaLas is tolerant of temperatures of 35°C but both CaLam and CaLaf are killed at this temperature [2, 15]. In addition to transmission by insects, the liberibacters can also be transmitted by vegetative propagation of infected budwood among rutaceous hosts. They can also be experimentally transferred to non-rutaceous hosts, such as periwinkle (*Catharanthus roseus*) [16], tomato (*Solanum lycopersicum*) [17], and tobacco (*Nicotiana tabacum*)[18], by dodder (*Cuscuta sp*.).

Liberibacters inhabit the sieve tube elements of phloem tissues in infected plants. In the past, detection of liberibacters mainly relied on biological assay [16], DNA probes [19], and transmission electron microscopy (TEM) [20]. More recently, conventional PCR [21–23] and quantitative PCR [24–26] and loop-mediated isothermal amplification (LAMP) [27] and LAMP combined with later flow detection [28] have been developed for the fast and accurate detection of liberibacters. All of these methods for the detection and quantification of the pathogen in plants and vectors rely on PCR-based methods, in which information on spatial and anatomical distributions is lost. Fluorescence *in situ* hybridization (FISH) is a powerful technique used to detect and localize the presence or absence of specific DNA sequences on chromosomes with fluorescent probes [29, 30]. FISH has been used to visualize and localize CaLas in psyllids and seed tissues using confocal laser scanning microscopy or TEM [11, 31]. It is worth noting that only 17 to 31% of CaLas cells were viable in samples assayed from HLB-symptomatic tissue, and DNA assays are not restricted to intact and viable cells [32–34], which are required for dissemination.

Tissue printing is used to determine cell-specific locations of macromolecules, such as proteins, enzymes, soluble metabolites or other antigens by labeling and visualization with the preservation of anatomical detail [35, 36]. The basic principle of tissue printing is that most of the cellular materials from a freshly cut surface can be easily transferred by simple contact to an adhesive or absorptive surface with little or no diffusion leaving a physical imprint with detailed anatomical information [37]. If compared to tissue fixation, embedding, sectioning, and microscopy, the resolution of tissue anatomical prints is relatively low, but it is a simple and inexpensive technique that is particularly useful with many samples. Immuno tissue printing can also preserve and reveal the distribution of particular proteins in relatively large tissues such as sections of fruits, seeds and stems or in the intact leaf [38, 39].

Immuno tissue printing has been widely used for the diagnosis and localization of viral, bacterial or fungal pathogens in plants [40–42]. Though CaLas has been visualized by TEM in small sections of tissue, the systematic distribution pattern of CaLas in citrus has only been determined by PCR-based methods [43, 44], in which anatomical detail is lost. The localization of CaLas in different tissues in plant hosts has not been reported except for studies by TEM [33]. *Citrus tristeza virus*, another phloem limited pathogen of citrus, has been detected using tissue printing combined with immuno detection in a Direct Tissue Blot Immunoassay (DTBIA) [45]. We demonstrated a DTBIA with nitrocellulose paper for the specific detection of CaLas in leaf petiole samples using anti-OmpA polyclonal antibodies (Ding et al., submitted).

Here we report a systematic histological localization of CaLas using DTBIA in freshly cut tissue sections. Different plant tissues, including citrus petioles, stems, roots, receptacles, peduncles and seeds as well as leaves of periwinkle plants were studied to illustrate the distribution pattern of CaLas in plant hosts. When compared to TEM, which is laborious, costly and time-consuming, the localization of CaLas with the DTBIA technique is simple to perform and no special equipment is needed. In addition to the low cost and scalable attributes of the DTBIA for detection of CaLas, the detailed spatial information on the distribution of CaLas within different plant organs may facilitate future research on the invasion and colonization of citrus by CaLas as well as host/pathogen interactions.

## Results

### Distribution of CaLas in sweet orange petioles

**Fig 1:**
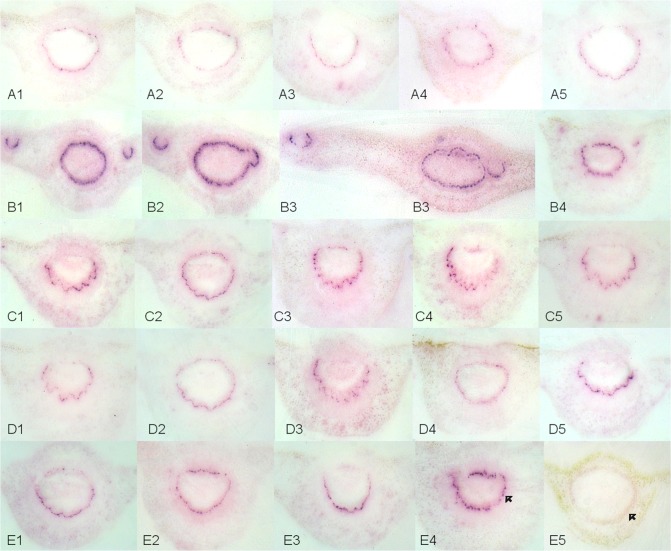
Localization and distribution of CaLas in sweet orange leaf midribs with blotchy mottling and zinc nutrient deficiency symptoms. Rabbit polyclonal anti-OmpA antibody (1:5000) and goat anti-rabbit secondary antibody (1:50,000) conjugated with alkaline phosphatase were used. A1-A5: CaLas in individual phloem sieve cells of leaf midribs with symptoms of zinc nutrient deficiency. B1-B4: CaLas in both phloem sieve cells of petioles and secondary veins of leaves with blotchy mottle symptoms. C1-E4: Uneven distribution of CaLas in phloem cells in serial sections of leaf midribs from leaves with blotchy mottle symptoms. E5: Healthy sweet orange. The arrow in panel E4 indicates CaLas infected individual phloem cells densely stained by the antibody reaction and the arrow in panel E5 indicates the lack of color localized in the phloem cells of the healthy control specimen.

The localization of CaLas within infected plant tissues from trees with leaves that showed blotchy mottling and zinc-nutrient deficiency symptoms was visualized using rabbit polyclonal antibodies raised against the outer membrane protein of CaLas. The outline and anatomical detail of the petiole sections was preserved in the tissue prints, and the phloem tissue was readily identifiable. Purple color, clearly restricted to phloem sieve tubes in the different sections of petioles, indicated the specific colonization of CaLas in HLB-infected phloem cells. No purple color was found in the phloem sieve tubes in the healthy controls (Fig 1). A diffuse and faint background color was sometimes seen in the tissue prints due to non-specific binding of the detection antibodies. Different petioles had different apparent concentrations of CaLas in the phloem sieve tubes (Fig 1A1–1A5 vs Fig 1B1–1B4), based on the intensity of color deposited on the membrane in the DTBIA. The apparent concentration of CaLas in the sieve tube elements was apparently higher in leaves with blotchy mottle than in leaves with symptoms of zinc chlorosis. The distribution of CaLas was not only in the petioles, but also in the secondary phloem sieve tubes of the winged petioles (Fig 1B1–1B4). Serial sections of same petiole were assayed. Although the tissue prints were all made from symptomatic petioles, the distribution of CaLas was uneven. Some sections produced very strong color in the phloem sieve tubes while other sections produced weaker color (Fig 1C1–1C5 vs Fig 1D1–1D5). In many cases color development, indicative of the presence of CaLas in the phloem sieve tubes, was much stronger in the lower half of the petioles (Fig 1A3, 1A5, 1C1, 1C4, 1D1, 1D3, 1D5, 1E1 and 1E3). However in other tissue prints, the distribution of CaLas in the phloem sieve tubes that encircled the petiole was more uniform (Fig 1A4, 1C2 and 1E4). The reason for this is not clear, but CaLas may begin to colonize petioles from the lower side of the leaf and only colonize the upper portion of the petiole as populations increase.

**Fig 2:**
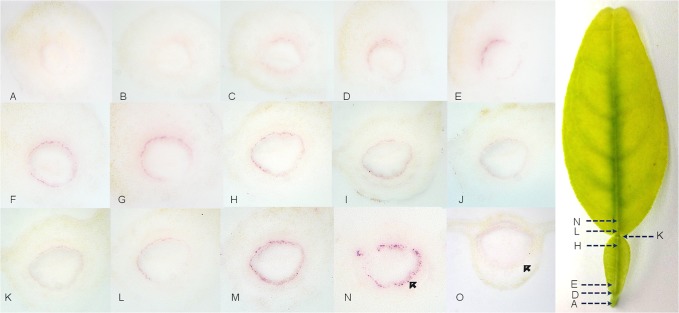
Localization and distribution of CaLas in a sweet orange leaf with yellowing symptoms. Samples were taken from the abscission zone (A) to the proximal portion of the midrib (N). A-D: Lower titer of CaLas in the sections of petioles close to the abscission zone between the leaf and the stem. E-H: higher titer of CaLas in petiole between the abscission zones. I-K: Lower titer of CaLas in the sections of petioles close to abscission zone between the leaf blade and the petiole. M-N: higher titer of CaLas in the proximal portion of the midrib. O: healthy sweet orange control, sampled at the midpoint between the abscission zones. The arrow in panel N indicates CaLas infected individual phloem cells densely stained by the antibody reaction and the arrow in panel O indicates the lack of color localized in the phloem cells of the healthy control specimen.

**Fig 3:**
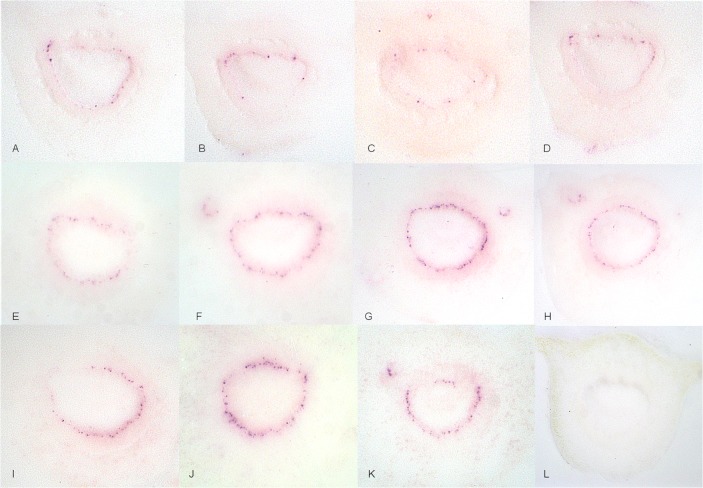
Irregular distribution of CaLas in phloem sieve cells. A-D: Localized infection of phloem cells by CaLas and E-K: generalized infection of phloem cells by CaLas. L: healthy sweet orange control, sampled at the midpoint between the abscission zones.

Fully developed leaf petioles from shoots with yellow leaf symptoms were serially sectioned to assess the distribution of CaLas in this tissue in more detail (Fig 2). The relative quantities of CaLas varied from section to section within the petiole. The sections taken from the petiole proximal to the stem had low levels of CaLas (Fig 2A–2C). In the main part of petiole, the concentration of CaLas was higher than in the part closest to the stem (Fig 2D–2H). Sections from I to N showed the distribution of CaLas around the leaf abscission area. At the end of petiole (Fig 2I–2K), the quantity of CaLas was lower compared to the main part of petiole. At the proximal portion of the midrib the titer of CaLas increased to higher levels (Fig 2L–2N). In many cases, the colonization of the phloem of petioles from symptomatic leaves was incomplete. In these cases some sieve tubes could be seen to contain large numbers CaLas, but adjacent phloem sieve tubes were empty (Fig 3).

### Distribution of CaLas in sweet orange stems

**Fig 4:**
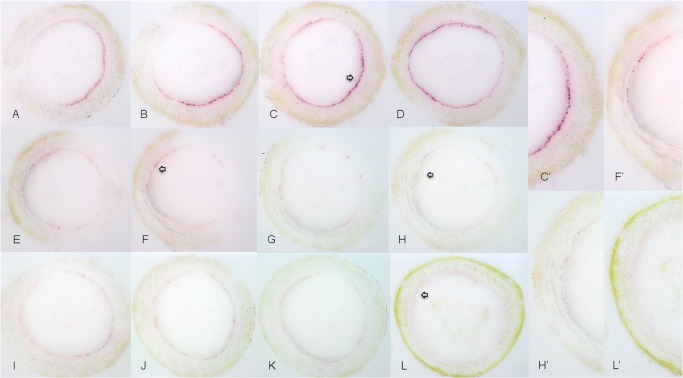
Localization and distribution of CaLas in sweet orange stem sections. A-D: CaLas in stem with symptomatic leaves. E-K: CaLas in stem with asymptomatic leaves. L: healthy sweet orange stem. A’, F’, H’ and L’ are higher magnifications of A, F, H and L.

The stem phloem plays an essential role in the transportation of nutrients. Serial sections of stems from trees infected with CaLas, but with symptomatic or asymptomatic leaves were prepared. Sections of stems with symptomatic leaves were extensively and relatively uniformly colonized by CaLas (Fig 4A–4D). Similar sections of stems that bore asymptomatic leaves had much less color that was also not uniformly distributed (Fig 4E–4K). No color developed in stem sections taken from healthy trees (Fig 4L). Enlargements of the tissue prints showed the pattern of colonization of stems by CaLas clearly (Fig 4C’, 4F’, 4H’ and 4L’). In stems that bore symptomatic leaves, the titer of CaLas was high and distributed in the phloem unevenly (Fig 4C’). In stems that bore asymptomatic leaves, the titer of CaLas was lower and distributed in the phloem very unevenly (Fig 4F’ and 4H’). In all sections tested, CaLas was found only in the phloem tissues as expected, and although often with an uneven distribution pattern, CaLas colonized the entire circle of phloem sieve cells along the stem. In all cases, purple color tightly localized in the phloem sieve tube; cells of CaLas infected samples could be easily distinguished from samples of healthy controls, though in rare cases there were nonspecific reactions with only a diffuse purple ring without tight localization in the phloem of healthy samples.

### Distribution of CaLas in the receptacle-peduncle region of sweet orange

**Fig 5:**
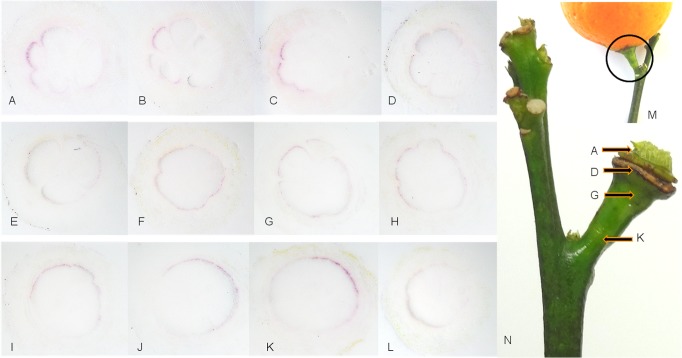
Localization and distribution of CaLas in sweet orange receptacle and peduncle. A-D: Calas in the receptacle; E-K: CaLas in the peduncle. L: healthy sweet orange control. N: Branch, and M: peduncle for orientation.

The peduncle and receptacle connect the stem and fruit. Because the sweet orange seed becomes colonized by CaLas, the pattern of CaLas in the peduncle and receptacle is also of interest. Serial sections from the receptacle to peduncle (Fig 5M) were prepared and DTBIA was carried out. CaLas was unevenly distributed in the receptacle (Fig 5A–5D). At the surface of the receptacle (Fig 5A) color was produced similar to the distribution in stems. In some sections (Fig 5B and 5C), the color development was similar but did not encompass the entire circle of phloem tissue. At the base of receptacle (Fig 5D), the apparent concentration of CaLas was very low, with only rare and weak purple spots. In subsequent sections of peduncle, stronger purple spots were produced (Fig 5E–5H), and at sections closer to the stem, stronger color reactions were obtained (Fig 5I–5K). Although the peduncle sections were continuous, the distribution of CaLas was not. In most receptacle and peduncle sections, though individual purple spots were observed in phloem cells, the relative concentration of CaLas was much lower as compared to sections tested from petioles (Figs 1 and 2) and stems with symptomatic leaves (Fig 4).

### Distribution of CaLas in sweet orange seed

**Fig 6:**
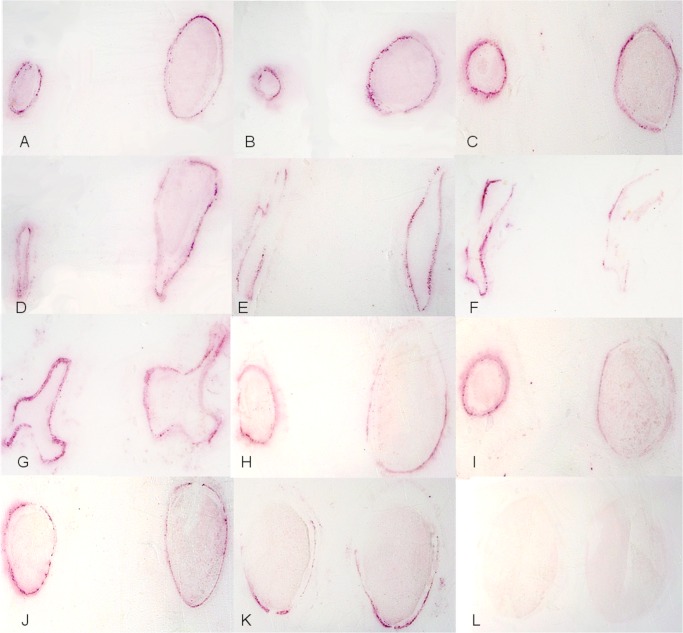
Localization and distribution of CaLas in sweet orange seed. A-G: CaLas infected and symptomatic ‘Valencia’ seeds collected from symptomatic fruit in Florida. H-K: Sweet orange seed from Florida (asymptomatic commercial fruit from supermarket). L: Healthy sweet orange seed.

Samples of seed were obtained from ‘Valencia’ sweet orange fruit (Florida) infected with CaLas and from healthy fruit. Two sections were prepared from each seed and the corresponding DTBIA images are presented side by side (Fig 6A–6K). For each pair of immuno tissue prints, the one on the left was made from the first section cut across the terminus of the seed bundle where it attached to the columnella, and the one on the right was a second cut distal to the first. Tissue prints (Fig 6A–6C) were cross sections of seeds from CaLas infected ‘Valencia’ sweet orange fruits with normal size and appearance, but harvested from a tree with strong symptoms of HLB (Florida). A high apparent concentration of CaLas was observed in the phloem vessels of the seed coats. Tissue prints (Fig 6D–6G) were prepared from sections of aborted seed with abnormal size and appearance. Very high apparent concentrations of CaLas were detected in those seeds. Tissue prints (Fig 6H–6I) were made from seed extracted from a sweet orange fruit without visible symptoms that originated in Florida and was purchased at a local supermarket. No color was observed in seed obtained from the healthy control fruit that was purchased locally and originated from California (Fig 6L). In all seed samples, CaLas was found only in the seed coat.

### Distribution of CaLas in roots of commercial sweet orange trees

**Fig 7:**
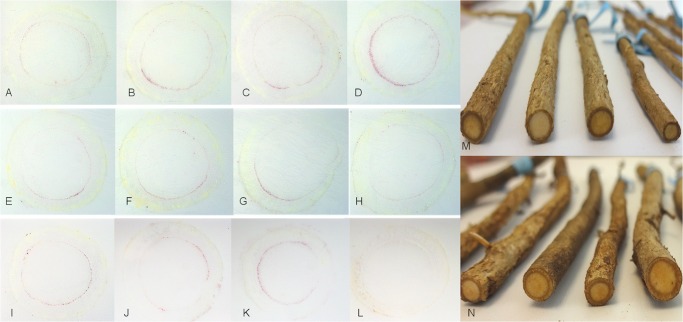
Localization and distribution of CaLas in citrus roots. A-H: CaLas in roots collected from HLB-symptomatic ‘Valencia’ orange tree 1. I-K: CaLas in roots collected from HLB-symptomatic ‘Valencia’ orange 2. L: Healthy sweet orange. M, N: Roots of ‘Valencia’ orange trees 1 and 2.

Sections of root samples (0.5–0.8 cm in diameter) collected from ‘Valencia’ sweet orange trees with typical symptoms of HLB were used to prepare DTBIA prints (Fig 7). In sample 1 (Fig 7A–7H) and sample 2 (Fig 7I–7K), CaLas was unevenly distributed in the randomly selected root samples from symptomatic trees. In some immuno tissue prints, relatively few bacteria were detected in the root phloem (Fig 7A and 7H), but these immuno tissue prints were easily distinguished from those of the healthy control (Fig 7L). DTBIA prints from other sections showed stronger reactions indicating more bacteria were present (Fig 7D and 7I–7K). Overall, compared to other tissue samples, such as petioles, stems and seeds, CaLas was more difficult to detect in the root sections. As in the other tissues tested, CaLas was present in phloem sieve tube cells but not in other vascular or surrounding tissues.

### Distribution of CaLas in periwinkle leaves

**Fig 8:**
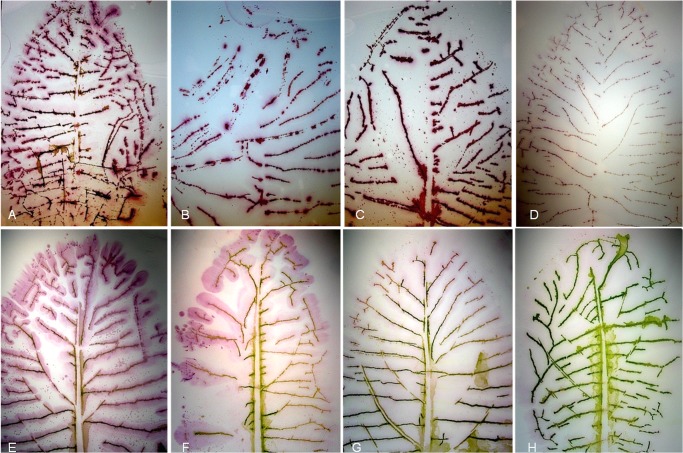
Localization and distribution of CaLas in whole leaves of periwinkle. A-G: DTBIA of leaves from CaLas-infected periwinkle plants. H: Healthy periwinkle.

**Fig 9:**
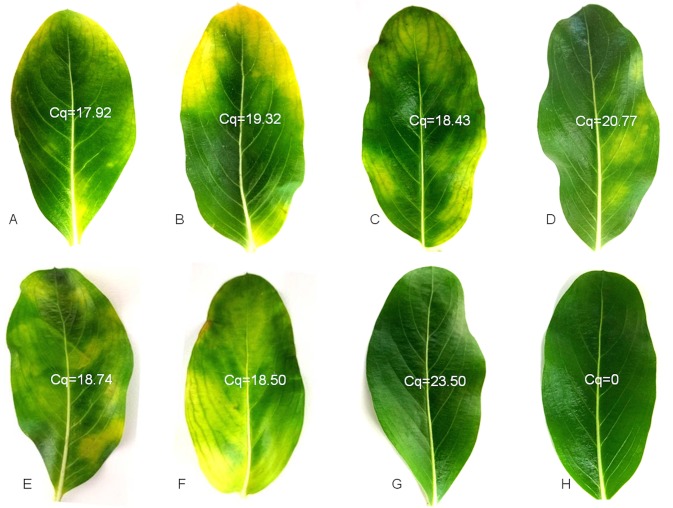
Visual symptoms in periwinkle leaves used for the DTBIA in Fig 8 with the Cq values for relative CaLas concentrations estimated by qPCR.

Madagascar Periwinkle (*Catharanthus roseus*), plays an important role in research on CaLas-host interactions. To investigate the distribution of CaLas, whole periwinkle leaves from CaLas-infected and healthy periwinkle were collected and printed onto nitrocellulose membranes. In infected leaves, CaLas was detected in the lateral leaf veins but not in the main vein (Fig 8A–8G). In some cases, the distribution of CaLas was throughout the leaf (Fig 8A–8E), but in other cases CaLas was found only in the most distal portions of the leaf veins (Fig 8F–8G). CaLas was not detected in the mesophyll of infected leaves, and there was no reaction from healthy periwinkle leaf control (Fig 8H). The presence of CaLas in the periwinkle leaves was also quantified by qPCR, and the results of the qPCR assays were consistent with the results of the immuno tissue prints (Fig 9). Leaves that produced the lowest Cq values (Fig 9A and 9C) also produced the strongest reactions in DTBIA prints (Fig 8A and 8C) and leaves with higher Cq values (Fig 9D and 9G) produced weaker signal in the DTBIA prints (Fig 8D and 8G).

### Specificity of the anti OmpA rabbit polyclonal antibody

The intense staining of CaLas in phloem cells of infected plants was completely blocked by pre incubation of the primary antibody with the antigen used to immunize the rabbit (S1 Fig). In other experiments the antibody was shown to not react with other vascular pathogens commonly found in citrus, including several phloem limited viruses and *Xylella fastidiosa* (Ding et al. in preparation).

## Discussion

Huanglongbing has been a serious disease in Asia for a century [2] but has been largely neglected until its introduction into the United States and Brazil [12]. In recent years, research on this disease has been aggressively pursued [46–49], and one area of focus has been related to molecular detection of the pathogen [25, 26, 44]. Despite vigorous efforts to obtain CaLas in culture, it is still a non-cultured bacterium [49, 50], which greatly hinders research compared to other well-characterized plant pathogenic bacteria. Knowledge of the detailed localization and distribution of pathogenic bacteria in plant tissues is essential for understanding bacterial colonization, mechanisms of pathogenicity, and dissemination to its insect vector.

Because CaLas DNA can be detected and quantified by PCR, the *in planta* distribution of CaLas DNA in different citrus organs including roots, midribs, leaf blades, petal, pistil, stamen, peduncle, fruit and bark has been quantified [43, 44]. However, all information on the detailed spatial distribution within plant tissues was lost during the extraction of DNA prior to PCR. Although transmission electron microscopy has provided high resolution and detailed information on the ultrastructural details of CaLas within individual cells, the technique does not scale well to the whole plant due to the labor and costs associated with sample preparation and the uneven distribution and relatively low titer of CaLas in its host [51, 52]. For these reasons the overall spatial distribution within different host tissues of infected citrus trees remains largely unknown.

CaLas was believed to be exclusively present in phloem sieve tube elements. In this study, CaLas was not detected in any other vascular or surrounding plant tissues, confirming that CaLas is limited to the phloem sieve tube elements. We also demonstrate an uneven pattern of distribution of CaLas within the phloem tissue (Fig 1). Whether or not CaLas forms biofilms with large numbers of cells in both newly colonized but asymptomatic and older symptomatic leaves is an open question [33, 51]. In our study, infected phloem sieve tube cells were both clustered together (Fig 1) and found separately (Fig 3). Such infected phloem sieve tubes were always observed in all kinds of tissues tested of CaLas-infected samples, whether symptomatic or asymptomatic samples were tested. Considering the size of CaLas cells (0.33 to 1.5 μm in diameter, 2.6 to 6.3 μm in length) [53], it is likely that visible purple spots observed in these immuno tissue prints are aggregates of CaLas. Thus the titer of CaLas in individual infected phloem sieve cells visualized in this study is likely to be high.

We have observed the colonization of sweet orange stem, receptacle and peduncle and seed coats by CaLas (Figs 4–7). Strong color development in the phloem cells of branches that bore symptomatic leaves indicated high titers of CaLas in stems (Fig 4). During fruit development, the peduncle-receptacle region increases and becomes stem-like, consisting of a woody cylinder surrounded by secondary phloem [54]. In DTBIA prints from the region of the receptacle and peduncle, CaLas was detected, and tissue prints closer to stem produced stronger color, suggesting a concentration gradient from stem to receptacle (Fig 5K–5D).

CaLas has been localized in seed previously, but the viability of these cells was unknown though they appeared to be intact when observed by TEM [11]. Here we used an anti-OmpA antibody. The presence of the OmpA protein target detected in DTBIA prints suggests the presence of functional OmpA antigens on viable cells. Therefore our results provide strong support that viable CaLas are present in these tissues. In the seeds, CaLas was distributed in the phloem sieve tube elements of the seed coat (Fig 6), which are directly connected to the receptacle of the fruit, but not to the nucellus. DTBIA prints made from the chalazal end of the seed produced consistent and strong color signal, and thus the seed are an excellent tissue for diagnostic testing.

The distribution of CaLas in roots from field samples (Florida) was also studied. As in other tissues, the colonization of roots by CaLas was detected, but the intensity of the color reaction tended to be much lower, and often produced weak signals (Fig 7). This is in accordance with the previous report that the concentration of CaLas was higher in leaves than in roots [32, 44]. Root samples collected from greenhouse trees with or without typical HLB symptoms were also repeatedly tested, and detection of CaLas in root tissues was less frequent than in petioles (Data not shown). Recent reports show that the qPCR assay used in these studies, HLBaspr [24] is more reliable when leaf samples, on which it was developed and validated are used, but less so when root samples are tested, because of cross reactions with an unknown bacterium [55, 56]. There is no doubt that CaLas can infect citrus roots, and it was reported that CaLas colonized roots before foliar symptoms developed [57] and CaLas was detected in fibrous roots of trees both with and without symptoms of HLB [56]. In the present study we prepared DTBIA prints from large roots, rather than from the feeder roots sampled by Kunta et al., and detection of CaLas in leaf and stem samples was more consistent than in DTBIA prints from root samples, especially when symptoms of HLB were present. This indicates a relatively lower concentration of CaLas in the roots than in the leaves, and is consistent with the idea that populations of CaLas are reduced in roots after foliar symptoms develop. It would be worthwhile to compare the PCR and qPCR methods tested [56] and the DTBIA described here on root samples.

Periwinkle leaves were used to establish the distribution of CaLas at the level of the whole leaf. CaLas colonized the veins of the leaves extensively, though in some cases the only detectable CaLas were at the distal ends of the veins. Although diffuse color was detected in the regions of the mesophyll of infected leaves (Fig 8), it was clearly caused by the forceful pressure used to make the tissue print, which squeezed the sap containing CaLas to the area surrounding the veins (Fig 8E and 8F). We do not interpret this diffuse color to indicate colonization of the mesophyll by CaLas. Note also that CaLas was not detected from the midribs of these leaves. CaLas was certainly present in these midribs, since it was present in the secondary veins (Fig 8A–8D), and the petioles from these leaves were tested by qPCR and found to contain CaLas Fig 9). We interpret this to mean that the structural integrity of the midribs was not destroyed during the preparation of the DTBIA prints, and so CaLas was not deposited on the membrane. Many attempts were made with citrus leaves to prepare similar DTBIA prints, but these were not successful, even when an electro transfer method was used. We interpret this to be related to the relative structural strength of the citrus leaf as compared with the periwinkle leaf.

A major portion of CaLas was in a non-viable state in symptomatic plant tissues [32, 34]. Most of our samples were clearly symptomatic with blotchy mottling, nutrient deficiency (Fig 1) and yellowing symptoms (Fig 2). These usually gave strong color reactions in the phloem sieve tubes as did samples from asymptomatic plants. However, colored spots in the phloem sieve tubes were much easier to observe from symptomatic samples. Although the portion of viable CaLas in samples with symptoms was low, symptomatic samples still contained sufficient viable CaLas to be detected. When samples from leaves with yellowing and zinc deficiency symptoms were tested, the color intensity in the phloem sieve tube cells tended to be lower as compared to samples from leaves with blotchy mottle symptoms (Figs 1 and 2). CaLas was difficult to detect in the abscission zones, but was readily detected in the proximal portion of the midrib (Fig 3).

## Conclusion

The results reported here demonstrate clearly that DTBIA can easily be used with an anti OmpA antibody to track viable CaLas in different plant tissues. The antibody is entirely specific for an epitope of the OmpA protein found on the surface of the bacterial cell. It allowed the detection, identification, and precise localization of the intact CaLas with a functional outer membrane antigen in all tissues tested. Because the outer membrane protein plays important roles in host-pathogen recognition, signal transduction and pathogenicity [58], anti-OmpA based immuno tissue prints may be useful for further research on CaLas-host interactions. Because DTBIA is also simple and inexpensive to implement, and can be readily scaled to any number of samples, it will also likely find application in diagnostic testing. It will be particularly useful as a supplement to qPCR testing protocols already in use (Ding et al., submitted).

## Material and Methods

### Plant Materials and Pathogens

Sweet orange (*Citrus sinensis* L.) trees infected with CaLas were propagated by bud inoculation and kept in the greenhouse. CaLas infected ‘Valencia’ sweet orange fruit and root samples were obtained from a commercial grove in Florida with permission of the owner. Sweet orange fruit were also purchased from a local supermarket (products of California and Florida). CaLas infected periwinkle (*Catharanthus roseus* L.) samples were propagated by branch graft inoculation to healthy periwinkle seedlings.

All plants were grown in Metro Mix 510 potting mix. The temperature of the greenhouse was maintained at 65–80 F (18–27°C) and ambient light was supplemented with high-pressure sodium vapor lighting on cloudy days and throughout the winter season to extend the photoperiod. Plants were watered as needed with water containing nitrogen/ phosphorus/ potassium (100/25/100 ppm), copper (2 ppm) and iron (6 ppm).

### Antibodies

A portion of the gene that encoded the major outer membrane protein (OmpA)of CaLas (YP_003065185) designated Omp3f was subcloned into the pET102/D-TOPO vector system (Life Technologies, Carlsbad, CA). The expressed protein fragment was purified by His/Ni chromatography (Life Technologies, Carlsbad, CA) under denaturing conditions and used for the preparation of polyclonal antibodies (Ding et al., submitted). A New Zealand white rabbit was immunized 4 times over the course of 56 days with 0.3 mg purified antigen mixed with an equal volume of TiterMax (Sigma Aldrich, St. Louis, MO) adjuvant per immunization (Cocalico Biologicals, Reamstown, PA). A commercial goat anti-rabbit polyclonal antibody (#12–448, EMD-Millipore, USA) conjugated with alkaline phosphatase was used as secondary antibody.

### Direct Tissue Blot Immunoassay procedure

CaLas-infected leaf, petiole, stem, root and fruit samples were obtained from commercial groves in Florida, or from the greenhouse. The corresponding healthy control samples were collected from the greenhouse. All samples were kept at 4°C before use. Seed samples were taken from fruit just before printing.

DTBIA was performed according to a published protocol [36] with minor modifications. Serial sections approximately 2 mm thick were cut from diseased or healthy tissues using separate razor blades. Samples were pressed for 10~15s onto nitrocellulose membranes with 0.22 μm pore size (Whatman).

DTBIA was also carried out with whole periwinkle leaves as described in a previous report [38]. In brief, individual periwinkle leaves were quickly immersed in liquid nitrogen and kept for 10–15s, then carefully removed with forceps, and placed onto nitrocellulose membranes. The undersurface of the leaf was in direct contact with the membrane and was allowed to return to room temperature for 5 min. Then the upper surface was covered with a waterproof paper and then was pressed evenly and firmly onto the membrane.

The printed membranes were air dried for 5–10 min at room temperature, and transferred to Phosphate Buffered Saline with 0.05% Tween-20 (PBST, pH7.4) and washed two times at room temperature (five minutes each) on a reciprocal shaker (80–100rpm) to reduce non-specific binding. Then the PBST was replaced with SuperBlock buffer (Thermo Fisher Scientific, Rockville, MD) in PBS and the membranes were incubated in SuperBlock buffer at room temperature for 2h. The membranes were then transferred to a standard blocking solution (PBST + 5% fat free skim milk) that contained the anti-OmpA polyclonal antibody at 5000 X dilution, incubated for 90 min at 37°C and then washed three times with PBST (10 min each). The secondary goat anti-rabbit polyclonal antibody conjugated with alkaline phosphatase was diluted 50,000 X, and incubated with the membranes for 1 h at 37°C to bind to the rabbit antibodies bound to the OmpA protein of CaLas. The tissue prints were washed three times with PBST (10 min each) before substrate was added (33 ul NBT + 16.5 BCIP) in 5 ml of alkaline phosphate assay buffer (Sigma Aldrich, St. Louis, MO). Incubation was stopped when color development could be seen (30 min—1h). All experiments were repeated in triplicate.

### Blocking of the primary rabbit polyclonal antibodies with the antigen used to immunize the antibodies

Tissue prints of CaLas infected and healthy petioles were prepared for DTBIA following the standard protocol above. The DTBIA was carried out as described above, or by using blocked primary antibody in the DTBIA. To prepare the blocked primary antibody the antibody was diluted 1:5000 in PBST + 5% fat free milk and mixed with 13.5 μg the antigen used to raise the antibody and incubated in a volume of 2 ml overnight at 4°C. The blocked antibody was then used in the DTBIA assay.

### Microscopy

All tissue prints were photographed with a Carl Zeiss Stereo Discovery V20 light microscope (Jena, Germany) equipped with a digital camera (AxioCamHR3).

### DNA extraction and qPCR

Sweet orange trees maintained in the greenhouse for sampling were assayed by qPCR to confirm that they were infected by CaLas. DNA was extracted from petioles and qPCR was performed as described [24]. The petioles of periwinkle leaves used for DTBIA were sampled and tested in the same way before the leaves were used to prepare tissue prints.

## Supporting Information

S1 Fig Binding of rabbit polyclonal antibodies against the OmpA epitope of CaLas in sweet orange petioles blocked by pre absorption with the antigen used to immunize the rabbit. **A**: DTBIA with rabbit polyclonal antibodies (1:5000) used as the primary antibody and goat anti-rabbit conjugated with alkaline phosphatase as the secondary antibody (1:50,000). CaLas-infected and healthy petioles are on the left and right, respectively. **B**: DTBIA as in A, except that the primary rabbit polyclonal antibody was incubated with 13.5 μg of the immunizing antigen prior to being used in the DTBIA.
(TIF)
