# Supplementary figures and images for: Localization and Distribution of 'Candidatus Liberibacter asiaticus’ in Citrus and Periwinkle by Direct Tissue Blot Immuno Assay with an Anti-OmpA Polyclonal Antibody
Source: PLoS One. 2015 May 6;10(5):e0123939. doi: 10.1371/journal.pone.0123939 (PMC4422590; doi:10.1371/journal.pone.0123939)

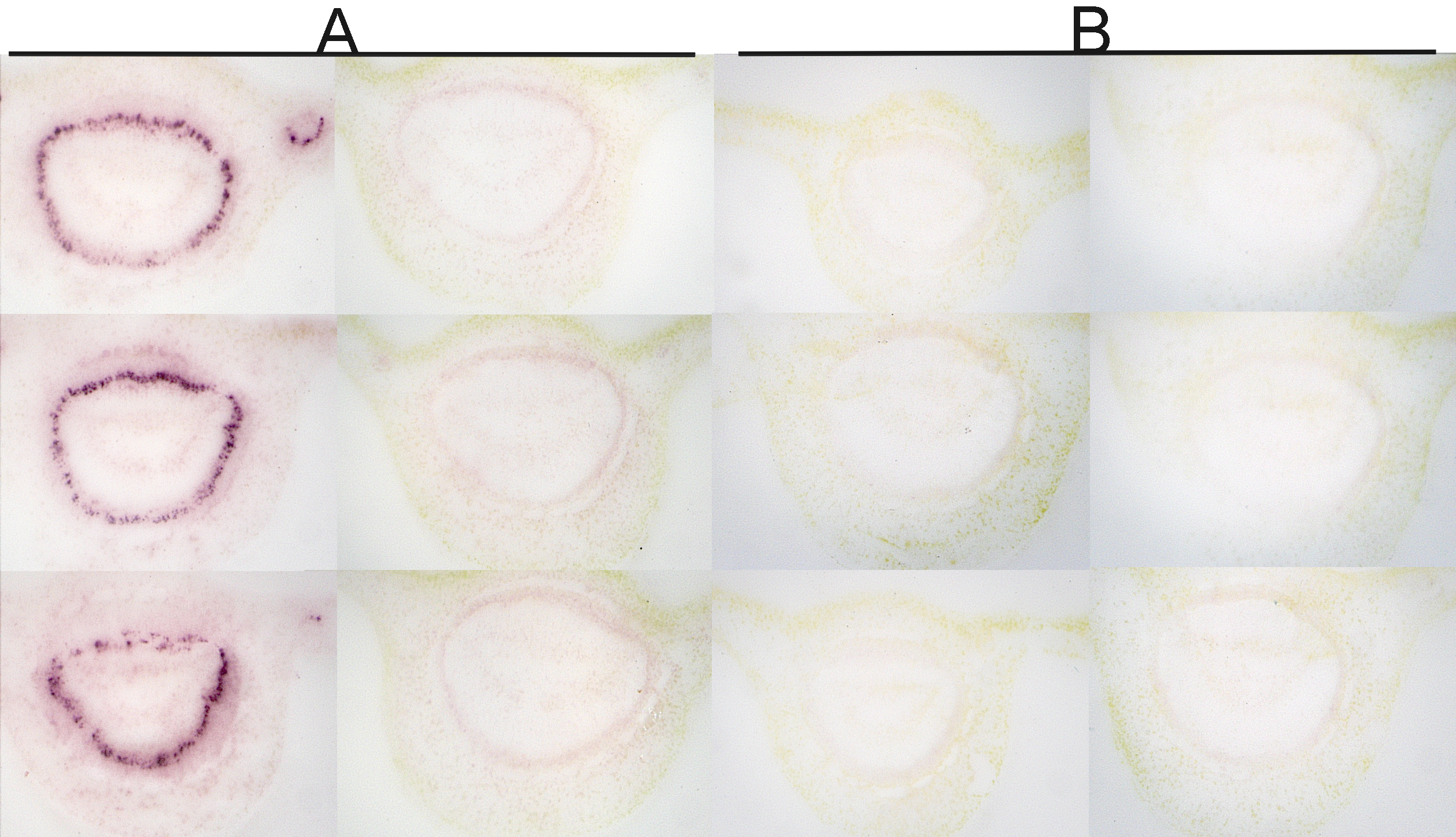

Supplement: S1 Fig — A: DTBIA with rabbit polyclonal antibodies (1:5000) used as the primary antibody and goat anti-rabbit conjugated with alkaline phosphatase as the secondary antibody (1:50,000). CaLas-infected and healthy petioles are on the left and right, respectively. B: DTBIA as in A, except that the primary rabbit polyclonal antibody was incubated with 13.5 μg of the immunizing antigen prior to being used in the DTBIA. (TIF) [file pone.0123939.s001.tif]
